# Vision-Based Detection and Classification of Used Electronic Parts

**DOI:** 10.3390/s22239079

**Published:** 2022-11-23

**Authors:** Praneel Chand, Sunil Lal

**Affiliations:** 1 Centre for Engineering and Industrial Design (CEID), Waikato Institute of Technology, Hamilton 3200, New Zealand; 2 School of Mathematical and Computational Sciences, Massey University, Palmerston North 4410, New Zealand

**Keywords:** vision system, object detection, object classification, shallow neural networks (SNNs), support vector machines (SVMs), deep learning, convolutional neural networks (CNNs)

## Abstract

Economic and environmental sustainability is becoming increasingly important in today’s world. Electronic waste (e-waste) is on the rise and options to reuse parts should be explored. Hence, this paper presents the development of vision-based methods for the detection and classification of used electronics parts. In particular, the problem of classifying commonly used and relatively expensive electronic project parts such as capacitors, potentiometers, and voltage regulator ICs is investigated. A multiple object workspace scenario with an overhead camera is investigated. A customized object detection algorithm determines regions of interest and extracts data for classification. Three classification methods are explored: (a) shallow neural networks (SNNs), (b) support vector machines (SVMs), and (c) deep learning with convolutional neural networks (CNNs). All three methods utilize 30 × 30-pixel grayscale image inputs. Shallow neural networks achieved the lowest overall accuracy of 85.6%. The SVM implementation produced its best results using a cubic kernel and principal component analysis (PCA) with 20 features. An overall accuracy of 95.2% was achieved with this setting. The deep learning CNN model has three convolution layers, two pooling layers, one fully connected layer, softmax, and a classification layer. The convolution layer filter size was set to four and adjusting the number of filters produced little variation in accuracy. An overall accuracy of 98.1% was achieved with the CNN model.

## 1. Introduction

One of the key principles of a circular economy [1] is the elimination of waste and pollution. This facilitates a robust system that is beneficial for businesses, humans, and the environment. Recycling and reusing products should be emphasized in every part of the economy. In educational environments where resourcing can be constrained, equipment and consumables used in projects can be recycled or reused [2].

**Figure 1:**
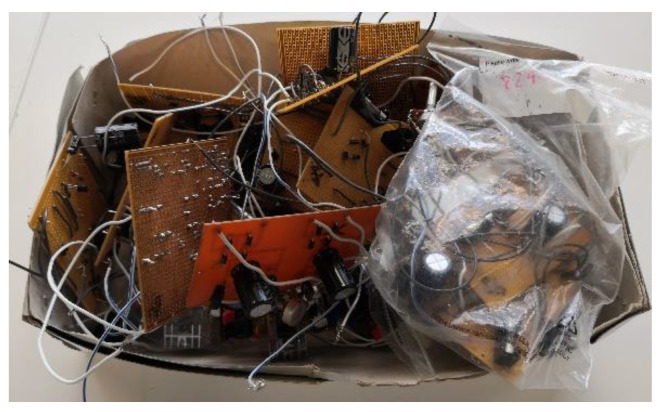
Circuit boards discarded after project work.

Higher education institutions that provide training for engineers often place high emphasis on practical activities and assessments. Courses in fields such as electrical and electronic engineering often rely on hardware components such as resistors, capacitors, inductors, voltage regulators, and diodes for project work. As an example, students are required to construct an electrotechnology product in the Electrical and Electronics Applications course at Waikato Institute of Technology [3]. The construction could be on a printed circuit board (PCB), Veroboard, or breadboard. After project work, the constructed PCBs are left in storage or thrown away (Figure 1). Used components are often discarded instead of being reused. In the circular economy concept, components on these circuit boards could be removed as part of soldering practice lessons. Since sorting parts manually is mundane, this could be achieved using an intelligent automated sorting system. Thus, this research proposes that a vision-based system be used to detect and classify parts.

According to Mathworks [4], identifying objects in images or videos is a computer vision technique known as object recognition. A variety of artificial intelligence methods can be used for object recognition. Techniques in machine learning and deep learning have become popular recently [5,6,7]. Object detection is similar to object recognition but varies in execution. In objection detection, instances of objects are identified and also located in an image. This enables many objects to be located and identified in an image.

Machine learning [8] is a sub-class of artificial intelligence and deep learning [9] is a sub-class of machine learning. Traditional machine learning approaches have interconnected steps such as segmentation, feature extraction and classification. Conventional traditional machine learning classification methods for object recognition include shallow neural networks (SNNs) and support vector machines (SVMs) [10,11]. Deep learning primarily utilizes deep neural networks that consist of multiple hidden layers. Feature extraction and classification is learned by the deep neural network. This provides superior flexibility because the framework can be re-trained using a custom dataset for transfer learning. Deep learning can also achieve better classification than traditional machine learning. However, it achieves this at the expense of requiring high-end computing power, larger training datasets, and longer training time. A comparison of traditional machine learning and deep learning applied to image recognition showed an increase in accuracy of less than 5% [12].

A common application of vision-based detection of electronic components is inspecting the integrity and quality of PCBs [13,14,15]. Image classification techniques based on deep neural networks have been used to detect integrated circuit (IC) components and verify their correct placement on the finished PCB product in [13]. Verification is similar to classification and a best accuracy of 92.31% was achieved. Machine learning is used to inspect components prior to assembly in [14]. The purpose of prior inspection is to reduce the number of defective components mounted and reduce falsely rejected components. Scale-invariant feature transform (SIFT) parameters are extracted from raw images and used with an artificial neural network (ANN) or an SVM for classification. Classification accuracies of up to approximately 97% were achieved. Tiny surface mount electronic components on PCBs are recognized using machine learning and deep learning in [15]. Machine learning with SVM+ principal component analysis (PCA) achieved an overall true positive rate (TPR) of 93.29%. The TPR was further improved to 99.999% with the deep learning-based Faster SqueezeNet.

Some recent methods to classify loose electrical and electronic components are based on deep learning models [16,17,18]. In [16], a customized CNN architecture is developed to classify three types of components: resistors, diodes, and capacitors. The developed system’s performance is benchmarked against pre-trained AlexNet, GoogleNet, ShuffleNet, and SqueezeNet deep learning architectures. While the accuracy of the pre-trained models ranged from 92.95% to 96.67%, the proposed CNN model achieved 98.99% accuracy. Post-training evaluation in a real-world setting was not conducted. In [17] and [18], variations of the ‘you only look once’ (YOLO) deep learning model [19] are utilized. The speed and accuracy of real-time object detection makes YOLO a popular choice. It is capable of directly outputting the position and category of an object through its neural network. Four electronic components (three types of capacitors and an inductor) are classified using YOLO-V3 and Mobilenet in [18]. A mean average precision (mAP) of 0.9521 was achieved. The YOLOv4-tiny network is combined with a multiscale attention module (MAM) and used to classify twenty types of electronic components in [17]. This improves the accuracy of the original algorithm from 93.74% to 98.6%. A potential deep learning model for detecting and classifying parts is Faster R-CNN [20]. However, the drawback of using Faster R-CNN for classifying electronic parts is explained in [13]. It achieves very poor results and according to the authors Faster R-CNN is not designed for small, relatively featureless objects such as ICs.

A non-deep learning based machine learning method for classifying electrical and electronic parts is presented in [21]. In this implementation, a K nearest neighbor (KNN) classification algorithm is used to classify capacitors, diodes, resistors, and transistors. Classification is performed based on physical appearances such as length, width, number of legs, shape (roundness of objects), and correlation of input images with standard database images. Full results and analysis are not presented, and accuracy is not quantified. While KNNs are simple and easy to implement, they can become significantly slower as the volume of data increases.

Recently, weakly supervised learning (WSL) has become popular in the computer vision community. A survey of various methods for object localization and detection is provided in [22]. An advantage of WSL is that it can perform object localization and detection at image level speeds of conventional fully supervised learning tasks. Typically, weakly labelled training images can be input to either machine learning methods (e.g, SVMs), or off-the-shelf deep models (e.g., AlexNet or R-CNN), or novel deep WSL frameworks. WSL is applied to video salient object detection in [23]. Co-salient object detection distinguishes common and salient objects in a group of relevant images. A summarize and search method that employs dynamic convolution to distinguish salient objects is presented in [24]. The current literature search did not determine any suitable implementations of WSL and video salient object detection for sorting electronic parts. 

A machine learning method that utilizes an SNN classifier to identify capacitors within a scene of scattered electronic components is presented in [25]. A feature extraction algorithm detects objects and converts them to a 20 × 20-pixel grayscale image for the SNN. An overall accuracy of 82.7% is achieved. This method is further extended to a three class problem for classifying capacitors, potentiometers, and voltage regulators in [26]. By increasing the size of the grayscale image to 30 × 30 pixels and correspondingly adjusting the size of the hidden neuron layer, an overall accuracy of 85.6% is achieved. Capacitor classification achieves an accuracy of 91.4%. 

Unlike the other reviewed methods [25,26], utilize lower resolution grayscale images for classification. This reduces the complexity of the classifier and requires lower computational power (processor and memory use). However, the accuracy is also reduced. Hence, this paper investigates the use of alternative methods based on SVM and CNN to improve classification using the low-resolution grayscale images.

## 2. Materials and Methods

### 2.1. Conceptual Framework

**Figure 2:**
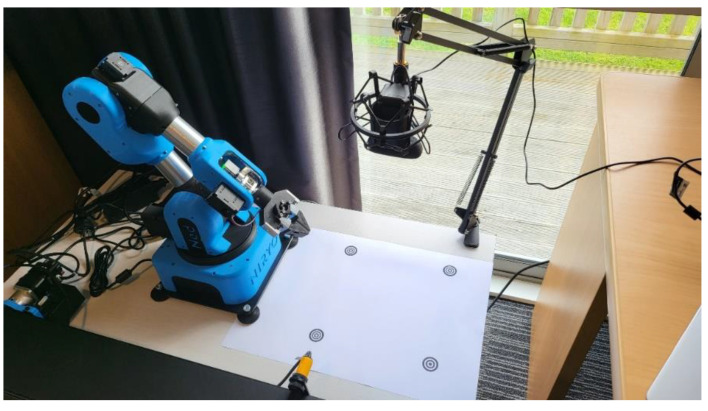
Visualization of object sorting system.

Figure 2 shows a visualization of the object sorting system. An overhead camera coupled with a Niryo Ned robotic arm [27] is used to detect, classify, and shift objects within a pre-defined workspace. This workspace has a size of 194 mm horizontally (h, x) by 194 mm vertically (v, y) and its boundaries are marked by one origin marker, top left (TL), and three edge markers, top right (TR), bottom left (BL), and bottom right (BR). The Niryo Ned robotic system has been selected because it features the open-source Robot Operating System (ROS) platform [28] and supports Matlab integration via the ROS Toolbox [29]. A graphical user interface (GUI)-based controller has been developed in Matlab to communicate commands to the robot and perform image acquisition [30]. Another feature is the relatively low cost of the hardware which is approximately US $3299 [30].

**Figure 3:**
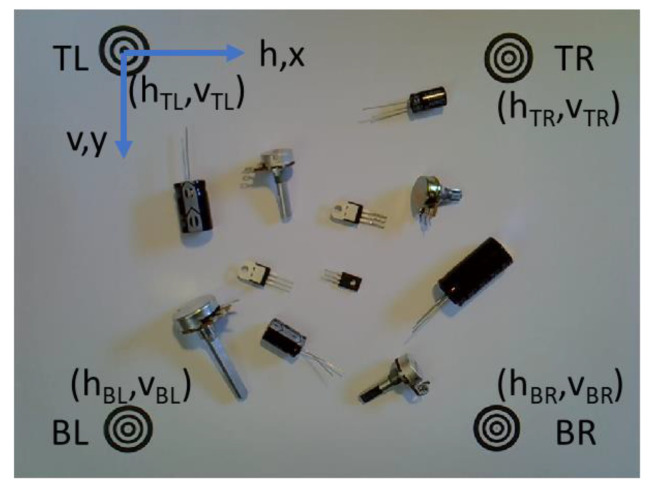
Sample camera image at the height of 0.37 m.

A Logitech HD C270 web camera is mounted in the center of the workspace. It is positioned at a height of approximately 0.37 m. Using a camera resolution of 960 × 720 pixels, the four boundary markers are clearly visible near the limits of the camera image at this height. Figure 3 illustrates a sample camera image at the height of 0.37 m. The camera height is adjustable since the four boundary markers are also used to automatically calibrate pixel distances (1), (2). The TL marker is the origin marker and is used to compute pixel and physical distances in the workspace. It also translates workspace distances to the robot’s reference frame.

x_cal_ = 194/(0.5(h_TR_ − h_TL_ + h_BR_ − h_BL_)), (1)

y_cal_ = 194/(0.5(v_BL_ − v_TL_ + v_BR_ − v_TR_)), (2)

where

x_cal_ is the x-axis calibrated pixel distance scale factor in mm/pixel,
y_cal_ is the y-axis calibrated pixel distance scale factor in mm/pixel.
H and v are horizontal and vertical pixel numbers, respectively.

**Figure 4:**
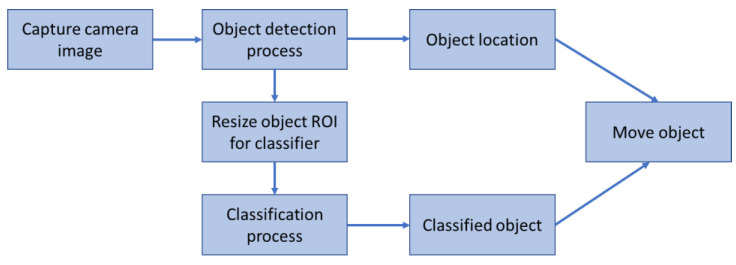
General framework of the vision-based classification system.

The overall general framework of the vision-based classification system designed in this paper is illustrated in Figure 4. First, an image of the workspace is captured using the web camera via Matlab. Following this, the acquired image is processed for object detection. Bounding boxes are placed around detected objects and the center of the bounding boxes represents the location (position) of the objects. After determining the bounding boxes, the partial image inside each bounding box is considered a region of interest (ROI) for the classifier and is resized according to the classifier requirements. Once the ROI is resized, the classifier uses it to match the image to an object class it has been trained to recognize. The classified object can then be moved by the robotic arm to a target location. 

### 2.2. Object (Component) Detection

**Figure 5:**
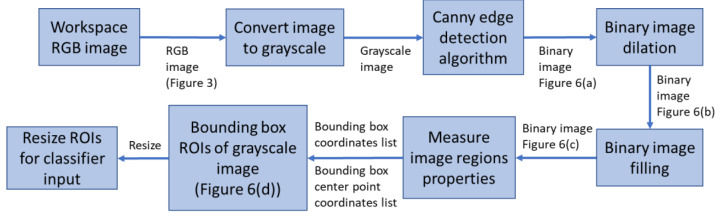
Main parts of the object detection process (Figure 3 and Figure 6).

**Figure 6:**
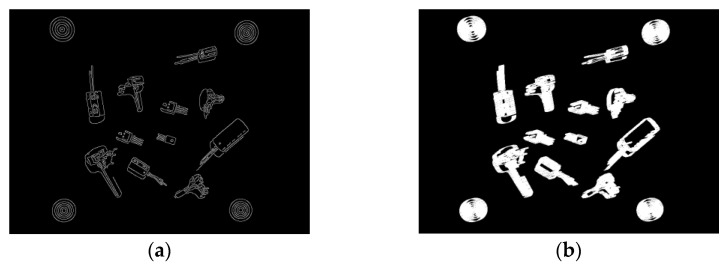

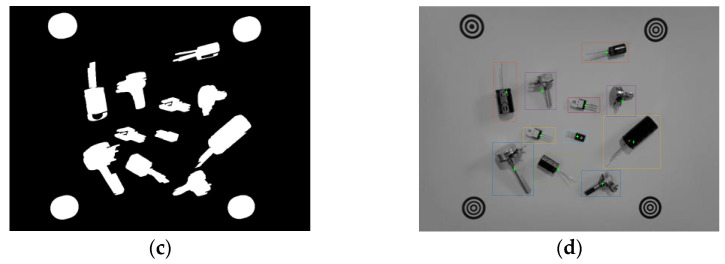
Sample images of various stages of the object detection process. (**a**) Canny edge detection binary image; (**b**) Binary image dilation; (**c**) Filled binary image; (**d**) Grayscale image with bounding boxes and center point coordinates.

The major parts of the object (component) detection process are highlighted in Figure 5. Various image processing algorithms are applied to extract ROIs containing unclassified objects. Figure 6 shows representative images of the various stages of the object detection process.

To reduce the complexity of the object detection and classification process, grayscale images are used. Hence, the first part of the process is to convert the RGB color image to grayscale using the weighted method (3). Following this, edge detection algorithms can be applied to determine the boundaries (outlines) of objects within images [31]. Of the available algorithms in Matlab (Sobel, Canny, Prewitt, and Roberts), Canny performed the best in detecting shape outlines (Figure 6a). Canny uses two thresholds which makes it less likely to be fooled by noise and more likely to detect true weak edges. The values for the high and low thresholds are 0.1 and 0.04, respectively.

gray = 0.299R + 0.587G + 0.114B.(3)

The output of the Canny edge detection algorithm is a binary image which is then dilated to further improve connectivity between the edges. This is achieved by applying a rectangular structuring element that enlarges the edges of the binary image (Figure 6b). Edge connectivity is important as the next stage involves flood-filling the binary image to form filled (solid) shapes representing the detected objects (Figure 6c). After flood-filling, the binary image is further processed by measuring the properties of the image regions. The “BoundingBox” property argument returns a set of positions and sizes of the smallest boxes, i, containing each detected object (Figure 6d) (4). This represents the ROIs. The green crosses in Figure 6d mark the bounding box centers (BBC) that represent the location of the objects in the workspace (5) and (6).

BB_i_ = [ho_i_, vo_i_, hw_i_, vh_i_], (4)

where

BB_i_ is the ith bounding box,
ho_i_ is the horizontal pixel number of the top left corner,
vo_i_ is the the vertical pixel number of the top left corner,
hw_i_ is the horizontal width in pixels,
vh_i_ is the vertical height in pixels.

BBC(h_i_,v_i_) = (ho_i_ + 0.5 × hw_i_, vo_i_, + 0.5 × vh_i_), (5)

BBC(x_i_,y_i_) = ((ho_i_ + 0.5 × hw_i_ − h_TL_) × x_cal_, (vo_i_,+ 0.5 × vh_i_ − v_TL_) × y_cal_). (6)

After detecting bounding boxes (ROIs), the size of each bounding box is checked against an estimated size threshold representing the dimensions of the smallest component to be detected. This eliminates small boxes that may have been erroneously detected due to noise or tiny holes in components such as voltage regulators. The pick and place task assumes that objects are physically separated and do not overlap.

The final stage before input to the component classifier involves standardizing the size of the ROI images. The ROIs of the grayscale image inside the bounding boxes are rescaled to 30 × 30 pixels. This has been arbitrarily selected to reduce complexity of the classifier and represents 900 inputs. 

### 2.3. Component Classification 

Component classification determines which class or category the detected component belongs to. Several methods of doing this are outlined in Section 1. Three techniques utilized in this research are described below.

#### 2.3.1. Shallow Neural Network (SNN)

**Figure 7:**
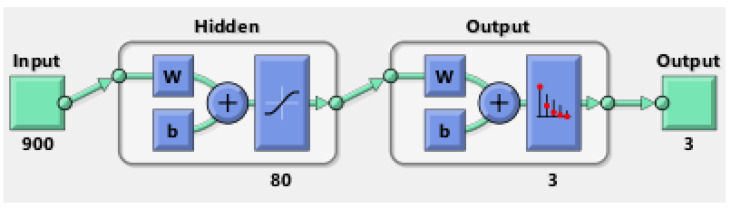
SNN architecture.

The SNN classifier has 900 inputs, one hidden layer, and three outputs representing the components (capacitor, potentiometer, and regulator) as shown in Figure 7. It is designed and implemented using the Neural Pattern Recognition tool (nprtool) in Matlab 2021a. The classifier is a feedforward neural network that is backpropagation trained using the scaled conjugate gradient method [32]. The performance function is the Cross-Entropy method (7) which generates batches of episodes and removes bad episodes in a batch to train the network on better ones. The tansig function is utilized in the hidden layer while the softmax function is employed in the output layer. These are the default settings of the nprtool. The main variable adjusted in the SNN is the number of neurons in the hidden layer.

(7) $$J=-\frac{1}{M}\sum_{m=1}^{M}\sum_{i=1}^{K}t_{m}^{i}\ln\left(y_{m}^{i}\right)$$

where

J is the cost,
M is the number of training data,
K is the number of output classes,
y is the output (contains K values, one for each class).

#### 2.3.2. Support Vector Machine (SVM) and Principal Component Analysis (PCA)

**Figure 8:**
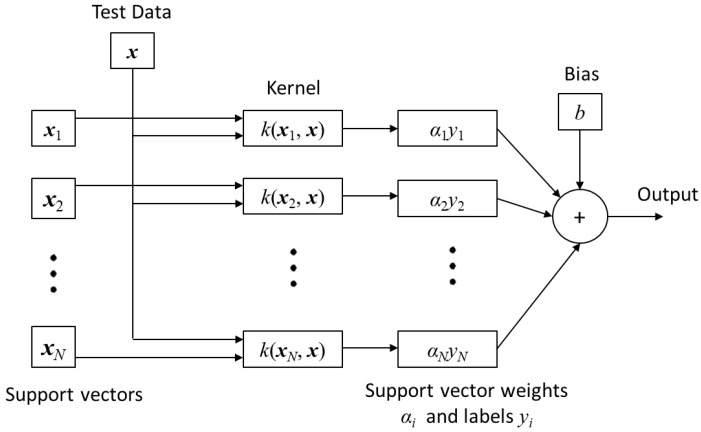
SVM architecture.

The SVM classifier also has 900 inputs and three outputs. It is designed using the Matlab Classification Learner App. Error-correcting output codes (ECOC) [33] are used to train the classifier which works by solving for a hyperplane that separates two class data with maximal margin [34]. The support vectors are the points which lie near the separating hyperplane. The SVM is trained for a 3-class problem on a one vs all approach. Since the original training data is not linearly separable, four different kernel functions K(**x**_i_,**x**) (linear (8), quadratic (9), cubic (10), and Gaussian (11)) are applied to the classifier. These transform the original input space into vectors of a highly dimensional feature space for the SVM to classify. The general structure of the SVM is shown in Figure 8.

(8) $$K\left(x_{i},x\right)=\left(x_{i}.x\right)$$

(9) $$K\left(x_{i},x\right)={\left(x_{i}.x+1\right)}^{2}$$

(10) $$K\left(x_{i},x\right)={\left(x_{i}.x+1\right)}^{3},$$

(11) K(xi,x)=e−‖xi−x‖22σ2.

Using 900 predictors in the input space can impair computational time. Hence, PCA [35] is used to determine the principal components for feature optimization [36,37]. This singular value decomposition reduces the data dimensionality and projects it to a lower dimensional environment for the SVM. This naturally comes at the expense of accuracy. Hence, it is important to compare the SVM + PCA classifier accuracy with the SVM only classifier.

#### 2.3.3. Convolutional Neural Network (CNN)

**Figure 9:**
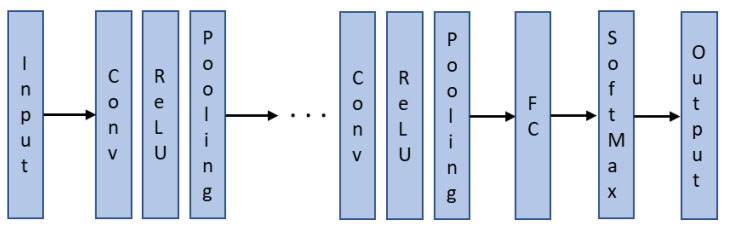
Basic structure of a CNN.

Like the SNN, the CNN classifier is also a feedforward neural network with 900 inputs (30 × 30-pixel image). It can extract features from the two-dimensional image and optimize parameters using backpropagation. The high performance of CNNs makes them a preferred deep learning architecture as outlined in Section 1 and in [38]. The basic structure of a CNN is shown in Figure 9. The hidden layers consist of a series of convolution, rectified linear unit (ReLU), and pooling layers. In the convolution layer, the image is examined by applying a filter smaller than the original image to determine its properties. Following this, the ReLU layer removes negative values from the output of the convolution layer. The pooling layer reduces the original size of the image by retaining important features and ignoring unnecessary features in the image. The fully connected (FC) layer converts the matrix image into a flat vector for the SoftMax function to determine the output classification.

**Figure 10:**
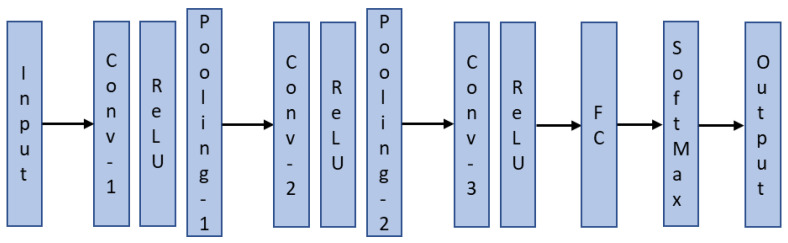
Architecture of the proposed CNN.

The architecture of the proposed CNN inspired by [39] has three convolution layers, two pooling layers, one fully connected layer, softmax, and an output classification layer as shown in Figure 10. The filter size for all three convolution layers is set to 4 × 4 with a stride of 1. A filter size of 3 × 3 is utilized for the two pooling layers and the stride is set to 3. 

## 3. Results

### 3.1. Datasets and Configuration

**Figure 11:**
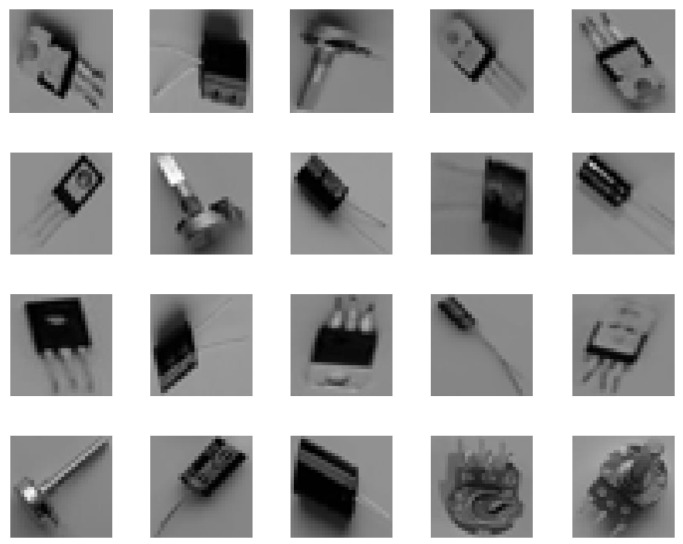
Sample of 25 images from the database.

The dataset used in this research consisted of a total of 1734 images extracted via the object detection process described in Section 2.2. Each class (capacitor, potentiometer, and regulator) had 578 images. A sample of the dataset images derived from object detection process is shown in Figure 11. Further details of the dataset are available in [40]. The dataset was randomly divided into 70% training (1214 images), 15% validation (260 images) and 15% test (260 images). Five-fold cross-validation was used in the training process. 

A Windows 10 HP ProBook 450 G7 laptop running Matlab 2021a was used to implement the various classifiers. The hardware configuration had an Intel i7-10510U processor and 16 GB RAM. 

After training the classifiers and testing them on the dataset, the best classifiers for SNN, SVM, and CNN were put to test in the real world. This was done with new independent data generated from the evaluation of ten multi-object scenes with a total of 104 objects. 

### 3.2. SNN Classifier Accuracy

**Figure 12:**
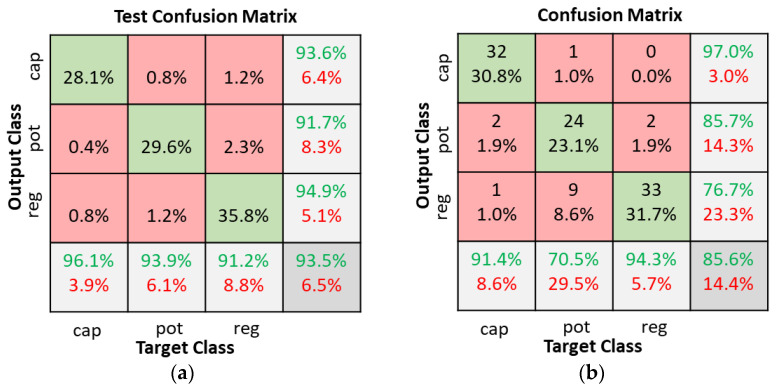
SNN classifiers with 80 hidden neurons confusion matrices. (**a**) Dataset test; (**b**) Real world test with 104 new objects.

The SNN classifier model was tested with a variety of hidden layer neurons ranging from 10 to 120. When the number of hidden neurons was below 40 (10, 20, or 30) the test accuracies were all below 90%. Details of the test accuracies when the number of hidden layer neurons varied between 40 and 120 is shown in Table 1. Good classification is possible with any of the classifiers with 40, 60, 80, or 100 neurons. The model with 80 hidden neurons was selected since it had the best overall accuracy. Figure 12 illustrates the test confusion matrix and the confusion matrix of the real-world test with 104 new objects. 

### 3.3. SVM + PCA Classifier Accuracy

**Figure 13:**
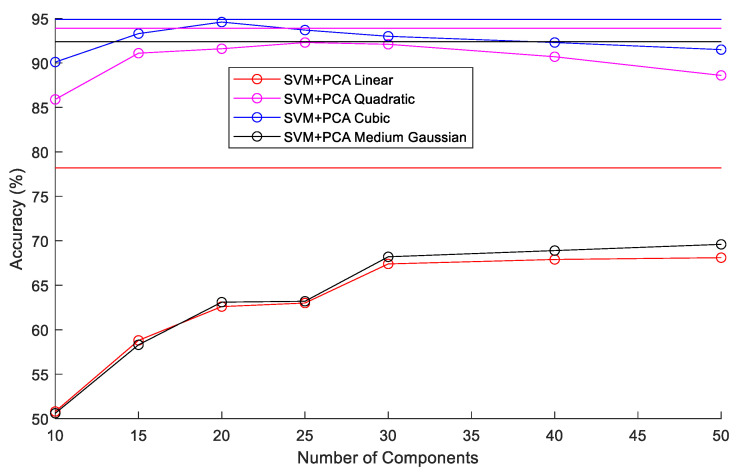
Comparison of SVM classifier accuracies with various kernels and PCA component numbers.

**Figure 14:**
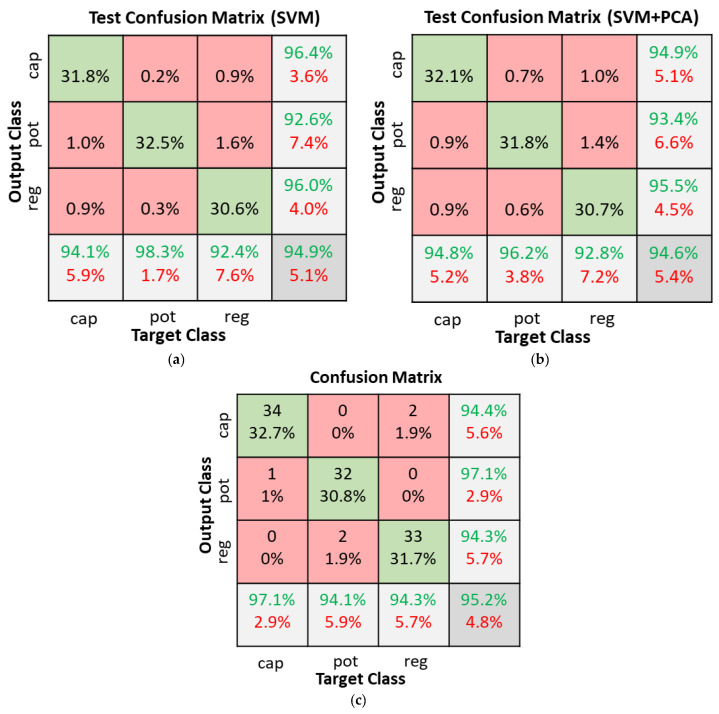
SVM classifiers with cubic kernel function confusion matrices. (**a**) SVM dataset test; (**b**) SVM + PCA with 20 components dataset test; (**c**) Real world test with 104 new objects.

In the SVM + PCA classifier experiments, the number of components in PCA was varied between 10 and 50. Four kernel functions (linear, quadratic, cubic, and medium Gaussian) were also tested. The results of the various combinations tested are summarized in Figure 13. The horizontal lines without markers in Figure 13 represent the accuracy of SVM classifiers using the various kernel functions without PCA. Without PCA, the SVM classifiers achieved accuracies of 78.2%, 93.9%, 94.9%, and 92.4% with the linear, quadratic, cubic, and medium Gaussian kernels, respectively. Using PCA with the linear and medium Gaussian kernels degraded accuracies to below 70%. The quadratic and cubic kernels achieved low reduction in accuracy when the number of PCA components was between 20 and 30. The SVMs with cubic kernel function were the best overall achieving accuracies of 94.9% without PCA and 94.6% with 20 component PCA. Figure 14 illustrates the test confusion matrix and the confusion matrix of the real-world test with 104 new objects for the SVM classifiers with cubic kernel function. The real-word test achieved the same results with the SVM and SVM + PCA with 20-component classifiers.

### 3.4. CNN Classifier Accuracy

**Figure 15:**
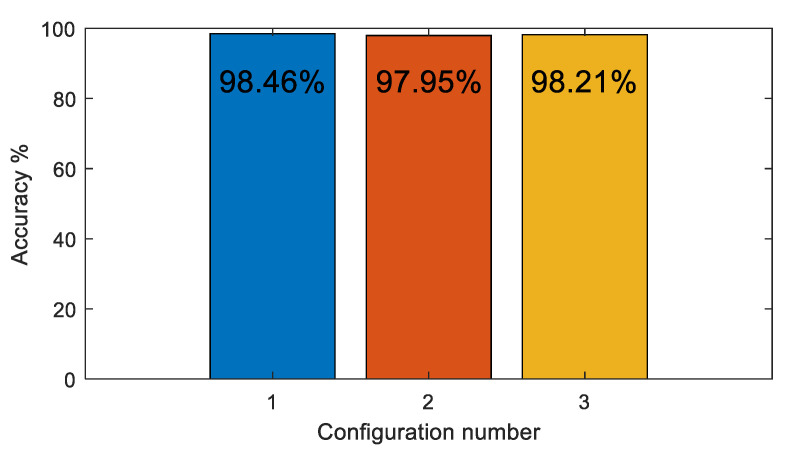
CNN classifier accuracy for various convolution filter configurations.

**Figure 16:**
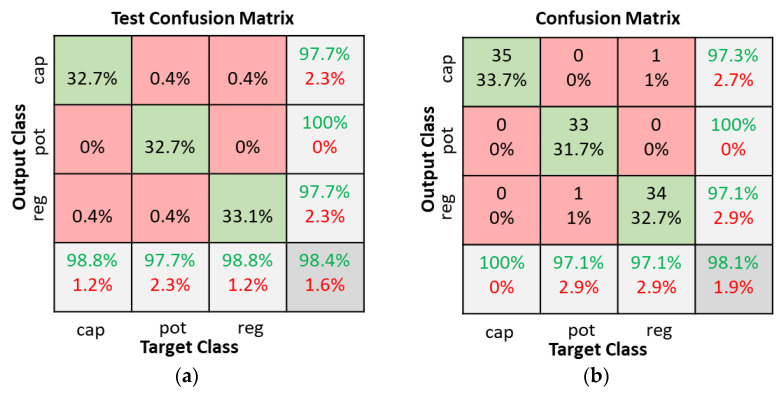
CNN classifiers with Configuration 1 confusion matrices. (**a**) Dataset test; (**b**) Real world test with 104 new objects.

The CNN classifier model was tested with a 4 × 4 filter size for all convolution layers and a 3 × 3 filter size for the pooling layers. The stride in the convolution and pooling layers was set to one and three, respectively. The number of filters in the convolution layers was varied as shown in Table 2. Table 3 shows the CNN model training parameters. As shown in Figure 15, there was little change in overall accuracy when the number of filters in the convolution layers varied. Hence, Configuration 1 was selected since it has the least number of filters. Figure 16 illustrates the test confusion matrix and the confusion matrix of the real-world test with 104 new objects for the CNN classifiers using Configuration 1.

## 4. Discussion

### 4.1. Overall Comparison of the Three Classifiers

**Figure 17:**
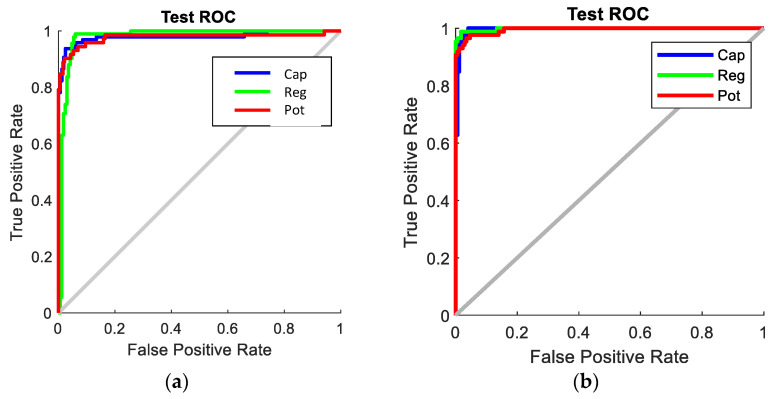

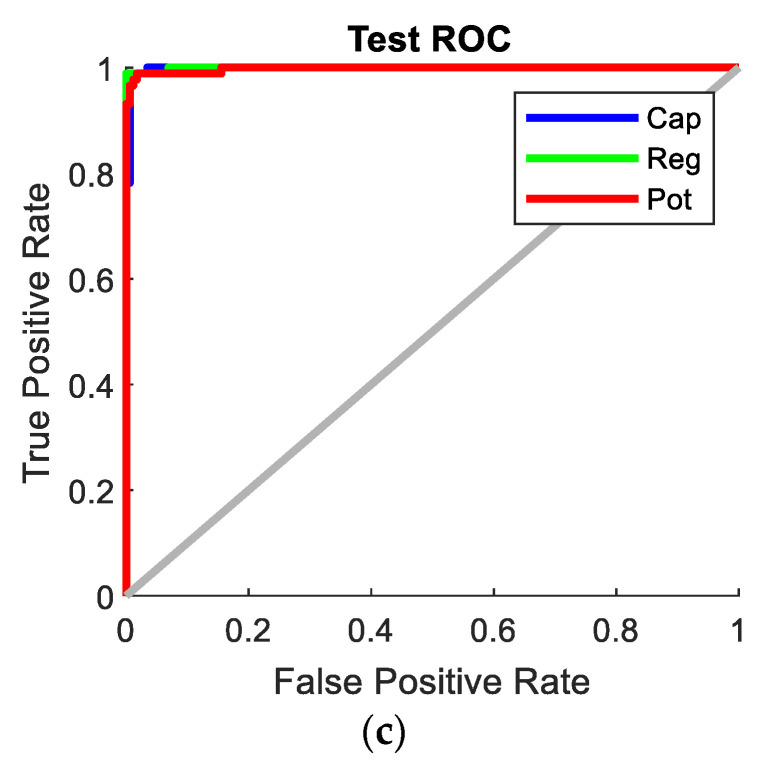
ROC curves of the best classifiers. (**a**) SNN with 80 hidden neurons; (**b**) SVM + PCA with 20 components; (**c**) CNN with Configuration 1.

**Figure 18:**
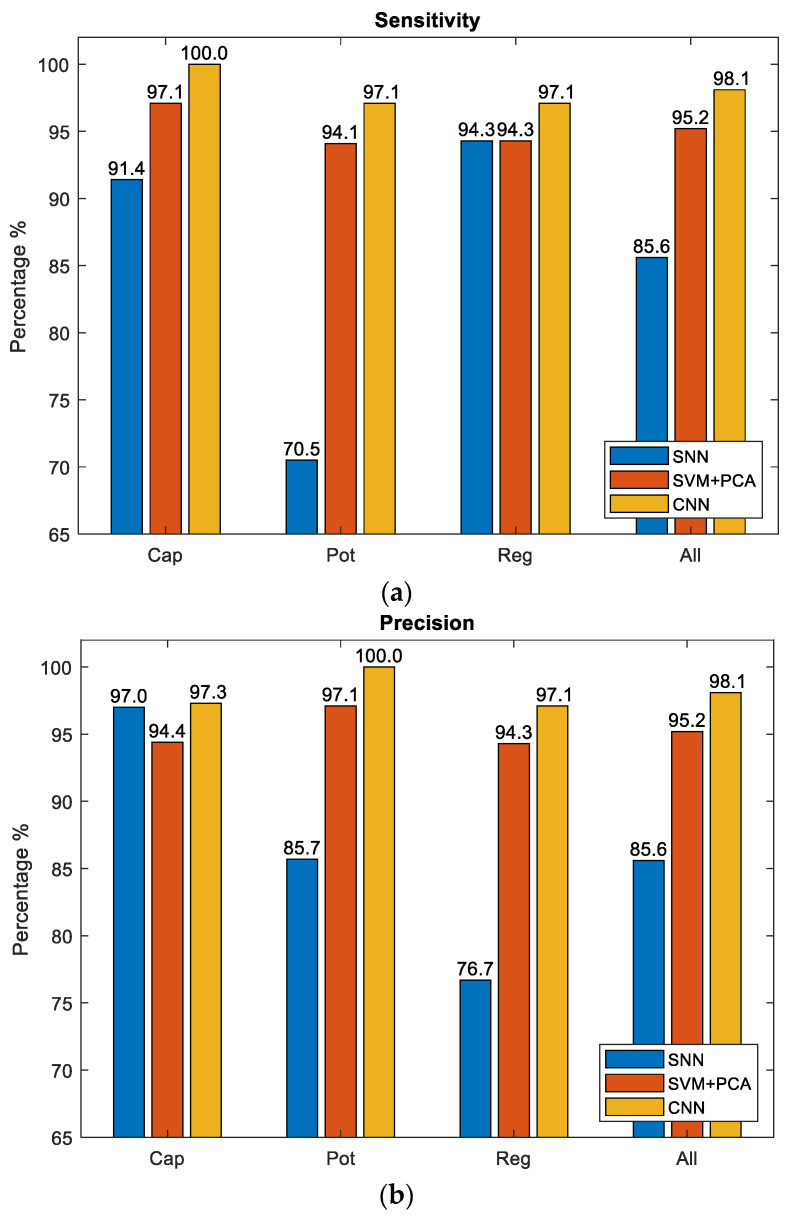
Key performance criteria metrics. (**a**) Sensitivity; (**b**) Precision.

The receiver operating characteristic (ROC) curves for the SNN with 80-hidden neuron classifier, SVM with cubic kernel and 20 PCA-component classifier, and CNN Configuration 1 classifier are shown in Figure 17. It is clearly visible that the CNN classifier has a superior ROC curve and performs the best for all object classes. Figure 18 compares the key performance criteria metrics of the classifiers based on the real-world test with 104 new objects. The CNN classifier has the best sensitivity and precision across all component classes. It also achieved the best accuracy of 98.1%. The SVM + PCA classifier can produce good results which are close to the CNN.

### 4.2. Comparison with Accuracy of Other Classifiers

The classifiers developed in this paper utilize low-resolution grayscale images. Other methods reviewed in Section 1 use higher resolution and color images. Therefore, these other classifiers are inherently more complex and require heavier computational power. Table 4 compares the CNN classifier presented in this paper with the properties of other representative deep learning models from Section 1. Model complexity excludes the ReLU layers for all models. A direct comparison of computation volume and speed is not possible due to variations such as image resolution and object class numbers. Therefore, an approximate comparison based on image input size and network complexity is made in Table 4. The key feature of our method is that it can perform on a standard laptop computer. The accuracy level of the developed CNN classifier is comparable with the other methods despite it using low resolution (30 × 30-pixel) grayscale images. However, the classifiers developed in [15,17] are capable of detecting a much wider range of electronic components. The training dataset employed in this research is small but sufficient for the three types of parts as there is not a large variation in physical properties of the items in each class. This is validated based on the classification results. The dataset can be expanded to include a larger variety of project parts if needed. For example, if ceramic and electrolytic capacitors need to be classified, then a new or expanded dataset can be utilized. The method presented in this paper is like YOLO as it has the ability to detect and classify electronic components with a single image of the entire workspace.

## 5. Conclusions

This paper presented the development of vision-based methods for the detection and classification of used electronic parts. Three classes of components were considered: capacitors, potentiometers, and voltage regulator ICs. A customized method for detecting multiple objects in a workspace and extracting data for classifier input was developed. Low resolution (30 × 30-pixel) grayscale images are input into the classifiers. This reduces the complexity of the classifiers and inherently requires lower computational power (processor and memory use). Three types of classifiers were investigated: SNN, SVM + PCA, and CNN. After training and testing the classifiers on the dataset, the best classifiers were put to test in the real world. As expected, the SNN classifier achieved lowest overall accuracy (93.5% in dataset and 85.6% in real word). This was followed by the SVM + PCA classifier with 20 components (94.6% in dataset and 95.2% in real world). The best accuracy was achieved with the CNN classifier (98.4% in dataset and 98.1% in real world). The accuracy of the CNN classifier is comparable to other relevant deep learning models.

Future work will involve extending this detection and classification method to other electronic parts. This will require increasing the dataset size for each component. The size of the input image to the classifier is currently limited by the resolution of the camera (960 × 720 pixels). In addition to this, the pick and place of objects detected via the developed object detection algorithm is being implemented. 

## Figures and Tables

**Table 1:** Accuracies of the tested SNN models.

Hidden Neurons	Test Accuracy %
40	93.1
60	92.3
80	93.5
100	92.3
120	87.8

**Table 2:** Filter numbers in convolution layers.

Configuration Number	Value [Conv-1 Conv-2 Conv-3]
1	[10 20 40]
2	[12 24 48]
3	[15 30 60]

**Table 3:** CNN model training parameters.

Parameters	Value
Optimize method	stochastic gradient descent with momentum (sgdm)
Initial learning rate	0.02
Maximum epochs	7
Validation frequency	20

**Table 4:** Comparison of the proposed CNN classifier with other deep learning methods.

Reference	Dataset Properties	Classes	Model Complexity	Accuracy
Atik (2022) [16]	Color, 227 × 227 × 3 pixels, 5332 images	3	Custom CNN with 13 layers	98.99%
Xu et al. (2020) [15]	Color, 112 × 112 × 3 pixels, 40000 images	22	Faster SqueezeNet with 23 layers	99.999% TPR when FPR = 10^−6^
Huang et al. (2019) [18]	Color, 416 × 416 × 3 pixels, 43,160 images	4	YOLO-V3-Mobilenet with 30 layers	95.21% mAP
Guo et al. (2021) [17]	Color, 608 × 608 × 3 pixels, 12,000 images	20	YOLOv4-tiny + MAM with 24 layers	98.6% mAP
Proposed CNN classifier	Grayscale, 30 × 30 pixels, 1734 images	3	Custom CNN with 7 layers	98.4% test, 98.1% real world test

## Data Availability

Datasets used in this study are available from Praneel Chand via email: praneelchand10@yahoo.co.nz.
